# Impact of perineal pain and delivery related factors on interference with activities of daily living until 1 month postpartum: A longitudinal prospective study

**DOI:** 10.1186/s12884-024-06618-5

**Published:** 2024-06-27

**Authors:** Akiko Yamada, Yuki Takahashi, Yurika Usami, Koji Tamakoshi

**Affiliations:** 1 Former Department of Integrated Health Sciences, Nagoya University Graduate School of Medicine, Nagoya, Japan; 2 Department of Integrated Health Sciences, Nagoya University Graduate School of Medicine, 1-1-20 Daiko-Minami, Higashi-ku, Nagoya, Aichi 461-8673 Japan

**Keywords:** Interference with activities of daily living, Perineal pain, Perineal injury, Postpartum

## Abstract

**Background:**

Interference with activities of daily living can negatively impact maternal practices both physically and psychologically. This study aimed to explore the patterns of interference with activities of daily living and perineal pain among Japanese women until 1 month postpartum. Furthermore, we aimed to describe how both perineal pain and delivery-related factors were associated with interference with activities of daily living.

**Methods:**

This study was part of a larger prospective longitudinal study conducted at five maternity hospitals in Japan. The participants were 293 women who had full-term vaginal deliveries and singleton infants. Participants self-evaluated their perineal pain and interference with activities of daily living using a 100 mm visual analogue scale and ‘behaviour that interferes with daily life scale’ at day 1, day 5, and 1 month postpartum. We used a linear mixed model to calculate the fixed-effects parameter estimates and their 95% confidence intervals. Interference with activities of daily living, which included difficulty sitting, difficulty moving, and difficulties with excretion and cleanliness, were set as the dependent variables.

**Results:**

The final analysis included 184 participants with a mean age of 31.5±4.5 years. Perineal pain and the three sub-scales of interference with activities of daily living reduced from day 1 to 5 postpartum, and further from day 5 to 1 month postpartum (perineal pain, *p*<0.01, *p*<0.01; difficulty sitting, *p*<0.01, *p*<0.01; difficulty moving, *p*<0.01, *p*<0.01; difficulties with excretion and cleanliness, *p*<0.01,* p*<0.01). These tendencies did not change, even adjusted for independent variables using a mixed model. In the mixed model for follow-up data, perineal pain was a significantly and positively associated with three sub-scales of interference with activities of daily living, even after adjusted for perineal injury and episiotomy.

**Conclusions:**

Positive relationships were observed between perineal pain and interference with activities of daily living until 1 month postpartum, although both reduced. To promote maternal role attainment through child-rearing since early postpartum, midwives should pay additional attention to mothers’ perineal pain as it could negatively affect their daily life and child-rearing.

## Background

Childbirth is an important life event that brings both joy and various challenges. Simultaneously, perineal discomfort is a common health issue experienced by women following vaginal delivery

It is well documented that perineal pain decreases over time, as well as does the interference with activities of daily living in those with perineal pain [1]. However, when postpartum perineal pain is severe, women experience a physical burden in their daily life and child-rearing activities [2, 3]. Furthermore, they also suffer from gaps between their ideal role as a ‘woman’ and ‘mother’ and the reality of their lives, which may have a negative psychological impact [4]. A previous study found that difficulty with baby-rearing activities was negatively correlated with maternal-infant attachment [5].

Macarthur and Macarthur [1] firstly reported the prevalence of interference with activities of daily living, such as sitting, urinating, walking, and sleeping, at day 1, 7, and 6 weeks postpartum according to the degree of perineal injury. Few women experienced interference with activities of daily living at 6 weeks postpartum. However, many women experienced interference at day1 and 7 postpartum, regardless of the degree of perineal injury. In addition, women without perineal injury also reported perceived interference with activities of daily living. Pereira et al. [6] investigated pain in women, such as perineal, abdominal, low back, and neck pain, and found that it caused their functional limitations, such as, sitting, getting up from a sitting position, walking, and lying down, both after a caesarean section and vaginal delivery in the immediate postpartum period. Takeuchi [7] developed a 24-item Japanese scale with four subscales to assess behaviours that interfered with daily life which was used to examine the association between maternal demographics, obstetric factors, and interference with daily life in Japan. Her study showed that interference with activities of daily living was associated with episiotomy, the degree of perineal injury, maternal age, and parity for Japanese women. Previous studies demonstrated an association between perineal pain and interference with activities of daily living and degree of perineal injury and interference with activities of daily living. Recently, limited studies have examined interference with activities of daily living. However, to our knowledge, no studies have reported an association between perineal injury and perineal pain and interference with activities of daily living.

World Health Organization reported that medical interventions in childbirth, such as, more analgesic deliveries and higher rates of caesarean sections, have increased rapidly [8]. Similarly in Japan, medical intervention in childbirth has been increasing owing to an increase in the number of high-risk pregnant women and infants [9]. Thus, we suspected that the number of women with perineal pain was also increasing. Consequently, the prevalence and intensity of perineal pain and interference with activities of daily living among women may have increased since the first study [1] was published.

Thus, this study aimed to examine the patterns of interference with activities of daily living and perineal pain among women until 1 month postpartum. In addition, we aimed to describe how both perineal pain and delivery-related factors were associated with interference with activities of daily living among Japanese women until 1 month postpartum.

## Methods

### Study design

This study was part of a larger longitudinal prospective study until 1 month postpartum [10].

### Setting and participants

This study was conducted from May to October 2018 and January to March 2020 at five maternity hospitals in Aichi, Japan.

We recruited pregnant women at approximately 36 gestational weeks from the outpatient ward. Inclusion criteria during pregnancy were women: 1) aged ≥20 years, 2) with singleton pregnancy, 3) regular antenatal maternity check-ups, and 4) could read and write in Japanese. Exclusion criteria included women: 1) with a history of mental health disorders or serious medical conditions, 2) pregnancy complications, and 3) planned to undergo a caesarean section. Pregnant women who met the inclusion criteria were potential participants. After they delivered healthy full-term infants, we carefully selected the final participants from the potential participants. Postpartum inclusion criteria were both women and infants who had no abnormalities. Meanwhile, exclusion criteria were 1) emergency caesarean section, 2) Apgar score of <8 at both 1 and 5 minutes after birth, and 3) women for whom rooming-in during hospitalization was not feasible because of their different practices from those who were rooming-in. Finally, 293 pregnant women who provided written informed consent were included.

### Measurements

Perineal pain intensity was self-rated using a visual analogue scale (VAS) that ranged from 0 to 100 mm, with scores of 0 and 100 that indicated ‘no pain’ and ‘worst pain possible’, respectively [11, 12]. Participants were asked to self-report whether or not they had used painkillers within 24 hours prior to completing the questionnaire.

Interference with activities of daily living due to perineal pain was measured using the ‘Behaviour that interferes with daily life scale’ [7]. Before the questionnaire was used for this study, we obtained permission to use these questions. The validated Japanese questionnaire comprised four sub-scales: difficulty sitting (six items), loss of volition (six items), difficulty moving (six items), and difficulties with excretion and cleanliness (six items). Responses were rated on a 4-point Likert scale (1: strongly disagree; 2: disagree; 3: agree; 4: strongly agree). We used the three subscales that represented interference with activities of daily living. In our study, the Cronbach's alpha of each sub-scale on day 1 postpartum was as follows: 0.92, 0.92, 0.89 and the total Cronbach's alpha was 0.96. The Cronbach's alpha of each sub-scale on day 5 postpartum was as follows: 0.95, 0.94, 0.91 and the total Cronbach's alpha was 0.97. The Cronbach's alpha of each sub-scale at 1 month postpartum was as follows: 0.92, 0.91, 0.90 and the total Cronbach's alpha was 0.95.

### Obstetric variables

Participants’ age, parity, height, pre-pregnancy weight, weight at admission for delivery, use of epidural analgesia during labour, duration of labour, mode of delivery, use of episiotomy, degree of perineal injury, gestational age, and infant’s birth weight were collected. Data were obtained from medical and midwifery records by the research midwife.

### Data collection

After delivery, the midwife as a research assistant assessed whether the potential participants fulfilled the selection criteria. After permission was obtained from the head nurse to visit the participants’ room at day 1 postpartum, the midwife as a research assistant visited the participants between 11 am and 4 pm and distributed the questionnaire. On day 5 postpartum, the midwife as a research assistant visited the participants in a similar manner and distributed the questionnaire again. Participants were distributed the 1 month postpartum questionnaire when they visited the hospital for their 1 month check-up. During hospitalization, we provided the participants a choice to return their questionnaires either to a collection box at the hospital or by mail to avoid any interference with their rest time, breastfeeding, and taking care of their infants. The questionnaires at 1 month postpartum were collected by mail.

### Statistical analysis

Continuous and qualitative variables were presented as mean ± standard deviation and numbers and percentages, respectively. Patterns of perineal pain and interference with activities of daily living, which included difficulty sitting, difficulty moving, and difficulties with excretion and cleanliness, were analysed. Without considering follow-up data, crude mean scores and standard deviations of perineal pain, difficulty sitting, difficulty moving, and difficulties with excretion and cleanliness were calculated for the three periods. Furthermore, differences between the scores across the three periods were compared using a one-way repeated measures analysis of variance with Bonferroni corrections. In addition, a linear mixed model that considered the continuous follow-up data was used to calculate estimated mean scores and standard errors. This allowed full use of the available data, controlled for internal correlation and potential confounding variables. Second, Pearson’s correlation coefficients was used to evaluate the strength of the monotonic association between interference with activities of daily living and the VAS score for perineal pain. Third, Pearson’s correlation coefficients was used to determine the correlation of the interference with activities of daily living and values in other factors. Student’s t-test was used to compare the two groups. One-way analysis of variance was used to compare the three groups. Bonferroni correction was used as a post-hoc test to compare multiple groups. We categorized women with perineal injuries, such as perineal tears and/or episiotomy, as ‘perineal injury’ and women with intact perineum as ‘no perineal injury’. Finally, to estimate the independent contribution of perineal pain and delivery-related factors associated with interference with activities of daily living during the postpartum period until 1 month, we calculated fixed-effects parameter estimates and their 95% confidence intervals using a linear mixed model. Interference with activities of daily living was set as the dependent variable. Statistical significance was set at *p*<0.05. All statistical analyses were performed using SPSS Statistics for Windows, version 27.0 (IBM, Japan).

## Results

**Fig. 1:**
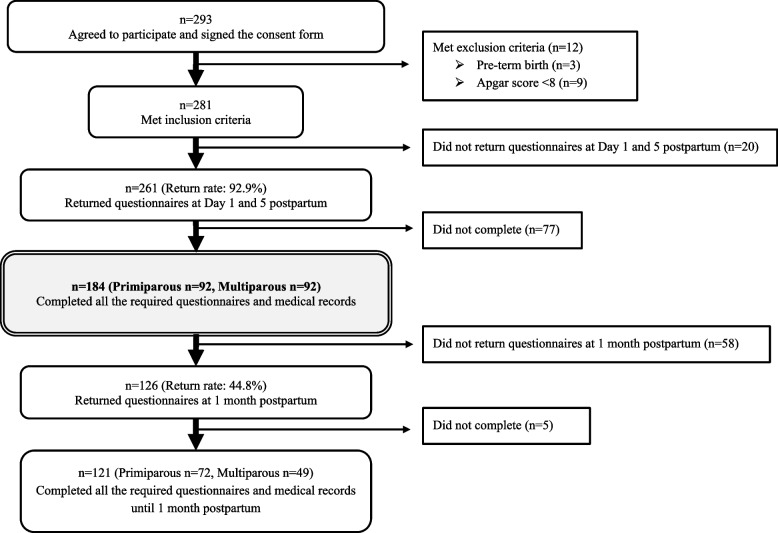
The flow chart of the participants

A total of 293 women were agreed to be participated and signed the consent form. In total, 261 women responded to the questionnaires and had their medical records collected (n=261/281, 92.9%). Of these, 77 women did not complete the questionnaire, left one or more questions unanswered, or had missing medical records. Finally, 184 women (n=184/261, 70.5%) returned fully completed questionnaires on day 1 and 5 postpartum. Of these, 121 (72 primiparous and 49 multiparous) women provided data for all the three periods (day 1, day 5, and 1 month postpartum). Others provided data for only two periods (day 1 and 5 postpartum). Figure 1 presents the flow chart of the participants.

Table 1 presents the participants’ characteristics. There were 92 (50.0%) primiparous women and 92 (50.0%) multiparous women, and their mean age was 31.5±4.5 years.

**Table 1:** Participants’ characteristics

	Total (*n*=184)	Primiparous (*n*=92)	Multiparous (*n*=92)
	Mean ± SD	Mean ± SD	Mean ± SD
Maternal age (years)	31.5 ± 4.5	30.3 ± 4.6	32.7 ± 4.0
Gestational age (days)	39w4d ± 7.2	39w4d ± 7.7	39w4d ± 6.6
Duration of labour (hours)	9.8 ± 9.0	13.6 ± 10.8	6.0 ± 4.1
Birth weight (gram)	3063.2 ± 362.1	2954.0 ± 334.7	3172.5 ± 357.1
	**n (%)**	** n (%)**	**n (%)**
Gestational weight gain based on pre-pregnancy BMI			
Below	34 (18.5)	19 (20.7)	15 (16.3)
Within	100 (54.3)	46 (50.0)	54 (58.7)
Above	50 (27.2)	27 (29.3)	23 (25.0)
Mode of delivery			
Spontaneous	161 (87.5)	73 (79.3)	88 (95.7)
Vacuum	23 (12.5)	19 (20.7)	4 (4.3)
Epidural analgesia			
Yes	15 (8.2)	6 (6.5)	9 (9.8)
No	169 (91.8)	86 (93.5)	83 (90.2)
Degree of perineal injuries			
Intact perineum	22 (12.0)	1 (1.1)	21 (22.8)
First-degree tear	18 (9.8)	2 (2.2)	16 (17.4)
Second-degree tear	141 (76.6)	86 (93.5)	55 (59.8)
Third-degree tear	3 (1.6)	3 (3.3)	0 (0)
Fourth-degree tear	0 (0)	0 (0)	0(0)
Episiotomy			
Yes	101 (54.9)	76 (82.6)	25 (27.2)
No	83 (45.1)	16 (17.4)	67 (72.8)
Day 1 postpartum; taking painkillers			
Yes	143 (77.7)	75 (81.5)	68 (73.9)
No	41 (22.3)	17 (18.5)	24 (26.1)
Day 5 postpartum; taking painkillers			
Yes	89 (48.4)	46 (50.0)	43 (46.7)
No	95 (51.6)	46 (50.0)	49 (53.3)
1 month postpartum; taking painkillers (*n*=121; primiparous, *n*=72, multiparous, *n*=49)			
Yes	3 (2.5)	2 (2.8)	1 (2.0)
No	118 (97.5)	70 (97.2)	48 (98.0)
1 month postpartum; types of neonatal nutrition (*n*=149; primiparous, *n*=76, multiparous, *n*=73)			
Breast milk	84 (56.4)	36 (47.4)	48 (65.8)
Combination / Formula	65 (43.6)	40 (52.6)	25 (34.2)

*SD* Standard Deviation, *BMI *Body Mass Index

Table 2 presents the patterns of VAS scores (0-100) for perineal pain and interference with activities of daily living of the subscale scores (6-24), which included difficulty sitting, difficulty moving, and difficulties with excretion and cleanliness, until 1 month postpartum. Perineal pain and interference with activities of daily living reduced from day 1 to 5 postpartum and further from day 5 to 1 month postpartum (perineal pain, *p*<0.01, *p*<0.01; difficulty sitting, *p*<0.01, *p*<0.01; difficulty moving, *p*<0.01, *p*<0.01; difficulties with excretion and cleanliness, *p*<0.01, *p*<0.01). These trends did not change after adjustment for independent variables using a mixed model.

**Table 2:** Patterns of perineal pain and interference with activities of daily living until 1 month postpartum

	Day 1 postpartum (D1)	Day 5 postpartum (D5)	1 month postpartum (M1)		
	Mean ± SD	Mean ± SD	Mean ± SD		
	Estimated mean ± SE	Estimated mean ± SE	Estimated mean ± SE	*p*-value	Post-hoc test
Perineal pain (*n*=121)^a^	48.2 ± 26.1	24.2 ± 22.4	2.2 ± 8.4	<0.01^*^	D1 > D5^**^, D5 > M1^**^
Perineal pain^b^	44.2 ± 1.7	22.0 ± 1.7	1.3 ± 2.0	<0.01^*^	D1 > D5^**^, D5 > M1^**^
Difficulty sitting (*n*=121)^a^	16.4 ± 5.0	12.0 ± 5.2	6.6 ± 2.0	<0.01^*^	D1 > D5^**^, D5 > M1^**^
Difficulty sitting^b^	15.8 ± 0.3	11.5 ± 0.3	6.3 ± 0.4	<0.01^*^	D1 > D5^**^, D5 > M1^**^
Difficulty sitting^c^	13.9 ± 0.5	11.6 ± 0.5	8.3 ± 0.6	<0.01^*^	D1 > D5^**^, D5 > M1^**^
Difficulty moving (*n*=122)^a^	17.8 ± 4.7	12.5 ± 5.3	6.7 ± 2.0	<0.01^*^	D1 > D5^**^, D5 > M1^**^
Difficulty moving^b^	17.0 ± 0.3	11.9 ± 0.3	6.4 ± 0.4	<0.01^*^	D1 > D5^**^, D5 > M1^**^
Difficulty moving^c^	14.9 ± 0.5	11.8 ± 0.5	8.0 ± 0.6	<0.01^*^	D1 > D5^**^, D5 > M1^**^
Difficulties with excretion and cleanliness (*n*=122)^a^	17.4 ± 4.3	13.9 ± 4.9	7.6 ± 2.9	<0.01^*^	D1 > D5^**^, D5 > M1^**^
Difficulties with excretion and cleanliness^b^	16.7 ± 0.3	13.2 ± 0.3	7.2 ± 0.4	0.01^*^	D1 > D5^**^, D5 > M1^**^
Difficulties with excretion and cleanliness^c^	14.8 ± 0.5	12.6 ± 0.5	7.7 ± 0.6	<0.01^*^	D1 > D5^**^, D5 > M1^**^

Score range: perineal pain; visual analogue scale (0-100), difficulty sitting (6-24), difficulty moving (6-24), difficulties with excretion and cleanliness (6-24)

*SD* Standard Deviation, *SE* Standard Error

^a^Crude mean and standard deviation. Difference among the three times after birth (day 1, day 5, 1 month postpartum) was evaluated using a one-way analysis of variance. Bonferroni correction was applied as a post-hoc test

^b^Estimated mean and standard error, calculated using a linear mixed model, the independent variable was the time after birth (*n*=184)

^c^Estimated mean and standard error, calculated using a linear mixed model, the independent variables were the time after birth, parity, mode of delivery, episiotomy, perineal injury, perineal pain and maternal age (*n*=184)

^*^ p<0.05, ^**^ p<0.01

Table 3 presents the factors associated with difficulty sitting on day 1, day 5, and 1 month postpartum. The VAS score for the perineal pain was significantly and positively associated with difficulty sitting both at day 1 and 5 postpartum (*p*<0.01, *p*<0.01). However, it was not significant at 1 month postpartum. In parity, primiparous women had significantly more difficulty sitting on day 1 and 5 postpartum (*p*<0.01, *p*<0.01). There was no significant difference in difficulty sitting among the gestational weight gain groups at three time points (*p*=0.25, *p*=0.22, *p*=0.37). Women who delivered by vacuum tended to have more difficulty sitting on day 1 postpartum (*p*=0.06) and significantly more difficulty sitting at day 5 postpartum (*p*=0.03). Women with perineal injury experienced significantly more difficulty sitting on day 1, day 5, and 1 month postpartum (*p*<0.01, *p*<0.01, *p*=0.02). In addition, women who had an episiotomy had more difficulty sitting on day 1 and 5 postpartum (*p*<0.01, *p*<0.01).

**Table 3:** Factors associated with difficulty sitting on day 1, day 5, and 1 month postpartum

	Day 1 postpartum		Day 5 postpartum		1 month postpartum	
	Correlation coefficient	*p*-value	Correlation coefficient	*p*-value	Correlation coefficient	*p*-value
Maternal age (years)^a^	-0.16	0.03^*^	-0.13	0.08	0.03	0.71
Gestational age (days)^a^	0.03	0.71	-0.001	0.99	0.06	0.51
Duration of labour (hours)^a^	0.22	<0.01^*^	0.10	0.16	0.10	0.29
Birth weight (gram)^a^	0.02	0.76	-0.07	0.35	0.07	0.42
Perineal pain (mm)^a^	0.55	<0.01^*^	0.63	<0.01^*^	0.05	0.60
	**Mean ± SD**		**Mean ± SD**		**Mean ± SD**	
Parity^b^						
Primiparous	18.0 ± 4.5		13.3 ± 5.4		6.7 ± 1.9	
Multiparous	13.6 ± 5.1	<0.01^*^	9.7 ± 4.5	<0.01^*^	6.6 ± 2.1	0.75
Gestational weight gain based on pre-pregnancy BMI^c^						
Below	14.9 ± 5.4		10.2 ± 4.5		6.6 ± 1.7	
Within	15.6 ± 5.4		11.6 ± 5.3		6.4 ± 1.6	
Above	16.7 ± 4.7	0.25	12.2 ± 5.5	0.22	7.0 ± 2.7	0.37
Mode of delivery^b^						
Spontaneous	15.5 ± 5.3		11.2 ± 5.2		6.5 ± 1.8	
Vacuum	17.7 ± 4.4	0.06	13.7 ± 5.3	0.03^*^	7.2 ± 2.7	0.35
Epidural analgesia^b^						
Yes	14.3 ± 6.5		10.3 ± 5.1		6.2 ± 0.6	
No	15.9 ± 5.1	0.24	11.6 ± 5.3	0.35	6.7 ± 2.1	0.49
Degree of perineal injuries^c^						
Intact perineum	10.7 ± 5.3		7.6 ± 3.0		7.2 ± 2.9	
First-degree tear	12.1 ± 4.7		7.9 ± 3.5		6.2 ± 0.4	
Second-degree tear	17.0 ± 4.7		12.4 ± 5.3		6.5 ± 1.9	
Third-degree tear	19.7 ± 2.1		16.0 ± 2.0		10.0 ± 4.0	
Fourth-degree tear		<0.01^*^		<0.01^*^		0.02^*^
Episiotomy^b^						
Yes	17.7 ± 4.2		13.1 ± 5.2		6.7 ± 2.1	
No	13.4 ± 5.5	<0.01^*^	9.5 ± 4.6	<0.01^*^	6.4 ± 1.8	0.45
Taking painkillers^b^						
Yes	16.2 ± 5.1		12.0 ± 5.3		6.7 ± 1.2	
No	14.4 ± 5.6	0.053	11.0 ± 5.2	0.18	6.6 ± 2.0	0.97
1 month postpartum; types of neonatal nutrition^b^						
Breast milk					6.8 ± 1.9	
Combination / Formula					6.5 ± 1.7	0.44

^a^Pearson’s correlation coefficients

^b^Student’s t-test

^c^One-way analysis of variance

SD: Standard Deviation; BMI: Body Mass Index

^*^ p<0.05

Table 4 presents the factors associated with difficulty moving on day 1, day 5, and 1 month postpartum. The VAS score for the perineal pain was significantly and positively associated with difficulty moving both at day 1 and 5 postpartum (*p*<0.01, *p*<0.01). However, it was not significant at 1 month postpartum. In parity, primiparous women had significantly more difficulty moving on day 1 and 5 postpartum (*p*<0.01, *p*<0.01). Women who delivered by vacuum had significantly more difficulty moving on day 1 postpartum (*p*=0.04). Women with perineal injury experienced significantly more difficulty moving on day 1, day 5, and 1 month postpartum (*p*<0.01, *p*<0.01, *p*<0.01). Women who had an episiotomy also had significantly more difficulty moving on day 1 and 5 postpartum (*p*<0.01, *p*<0.01). Women who took painkillers had significantly more difficulty moving on day 1 postpartum (*p*=0.01).

**Table 4:** Factors associated with difficulty moving on day 1, day 5, and 1 month postpartum

	Day 1 postpartum		Day 5 postpartum		1 month postpartum	
	Correlation coefficient	*p*-value	Correlation coefficient	*p*-value	Correlation coefficient	*p*-value
Maternal age (years)^a^	-0.11	0.15	-0.12	0.09	0.05	0.60
Gestational age (days)^a^	-0.002	0.98	-0.05	0.52	0.04	0.68
Duration of labour (hours)^a^	0.13	0.07	0.04	0.54	0.07	0.41
Birth weight (gram)^a^	-0.01	0.85	-0.08	0.31	0.02	0.81
Perineal pain (mm)^a^	0.57	<0.01^*^	0.59	<0.01^*^	0.14	0.12
	**Mean ± SD**		**Mean ± SD**		**Mean ± SD**	
Parity^b^						
Primiparous	18.9 ± 4.4		13.6 ± 5.4		6.7 ± 1.8	
Multiparous	15.0 ± 5.2	<0.01^*^	10.2 ± 4.4	<0.01^*^	6.6 ± 2.2	0.79
Gestational weight gain based on pre-pregnancy BMI^c^						
Below	16.7 ± 4.9		10.8 ± 4.6		6.7 ± 2.0	
Within	16.4 ± 5.3		11.8 ± 5.6		6.6 ± 1.7	
Above	18.4 ± 4.9	0.08	13.0 ± 4.7	0.13	6.8 ± 2.4	0.90
Mode of delivery^b^						
Spontaneous	16.7 ± 5.2		11.7 ± 5.1		6.6 ± 1.9	
Vacuum	19.0 ± 4.1	0.04^*^	13.9 ± 5.7	0.052	7.1 ± 2.4	0.41
Epidural analgesia^b^						
Yes	15.6 ± 6.2		10.1 ± 4.2		6.1 ± 0.3	
No	17.1 ± 5.1	0.29	12.1 ± 5.3	0.16	6.8 ± 2.0	0.32
Degree of perineal injuries^c^						
Intact perineum	11.6 ± 5.2		7.8 ± 3.8		7.3 ± 3.3	
First-degree tear	14.6 ± 5.3		8.1 ± 3.2		6.1 ± 0.3	
Second-degree tear	18.0 ± 4.5		13.0 ± 5.1		6.6 ± 1.8	
Third-degree tear	21.3 ± 3.1		16.3 ± 3.8		10.7 ± 4.5	
Fourth-degree tear		<0.01^*^		<0.01^*^		<0.01^*^
Episiotomy^b^						
Yes	18.6 ± 4.2		13.4 ± 5.2		6.8 ± 2.1	
No	15.0 ± 5.6	<0.01^*^	10.1 ± 4.7	<0.01^*^	6.6 ± 1.8	0.54
Taking painkillers^b^						
Yes	17.5 ± 4.8		12.5 ± 4.7		6.7 ± 0.6	
No	15.0 ± 5.8	0.01^*^	11.4 ± 5.6	0.18	6.7 ± 2.0	0.97
1 month postpartum; types of neonatal nutrition^b^						
Breast milk					6.8 ± 2.0	
Combination / Formula					6.5 ± 1.6	0.39

^a^Pearson’s correlation coefficients

^b^Student’s t-test

^c^One-way analysis of variance

SD: Standard Deviation; BMI: Body Mass Index

^*^ p<0.05

Table 5 presents the factors associated with difficulties with excretion and cleanliness on day 1, day 5, and 1 month postpartum. The VAS score for the perineal pain was significantly and positively associated with difficulties with excretion and cleanliness both at day 1 and 5 postpartum (*p*<0.01, *p*<0.01). However, it was not significant at 1 month postpartum. In parity, primiparous women had significantly more difficulties with excretion and cleanliness on day 1 and 5 postpartum (*p*<0.01, *p*<0.01). Women who delivered by vacuum had significantly more difficulties with excretion and cleanliness on day 1 postpartum (*p*=0.02). Women with perineal injury experienced significantly more difficulties with excretion and cleanliness on day 1 and 5 postpartum (*p*<0.01, *p*<0.01). Women who had an episiotomy also had significantly more difficulties with excretion and cleanliness on day 1 and 5 postpartum (*p*<0.01, *p*<0.01).

**Table 5:** Factors associated with difficulties excretion and cleanliness on day 1, 5, and 1 month postpartum

		Day 1 postpartum				Day 5 postpartum			1 month postpartum			
		Correlation coefficient		*p*-value		Correlation coefficient	*p*-value		Correlation coefficient			*p*-value
Maternal age (years)^a^		-0.02		0.83		-0.03	0.65		0.01			0.88
Gestational age (days)^a^		-0.02		0.76		0.004	0.95		0.05			0.62
Duration of labour (hours)^a^		0.14		0.06		0.06	0.39		0.05			0.59
Birth weight (gram)^a^		-0.08		0.31		-0.09	0.22		0.01			0.92
Perineal pain (mm)^a^		0.45		<0.01^*^		0.51	<0.01^*^		0.06			0.51
		**Mean ± SD**				**Mean ± SD**			**Mean ± SD**			
Parity^b^												
Primiparous		18.2 ± 3.8				14.9 ± 4.6					7.8 ± 3.0	
Multiparous		15.2 ± 4.6		<0.01^*^		11.6 ± 4.5	<0.01^*^				7.2 ± 2.8	0.22
Gestational weight gain based on pre-pregnancy BMI^c^												
Below		15.7 ± 4.6				12.0 ± 4.0					6.9 ± 2.4	
Within		16.7 ± 4.4				13.4 ± 5.1					7.7 ± 2.9	
Above		17.4 ± 4.6		0.25		13.7 ± 4.7	0.26				7.9 ± 3.3	0.43
Mode of delivery^b^												
Spontaneous		16.5 ± 4.6				13.0 ± 4.8					7.5 ± 2.9	
Vacuum		18.3 ± 3.2		0.02^*^		14.7 ± 4.5	0.12				7.9 ± 3.3	0.63
Epidural analgesia^b^												
Yes		17.3 ± 4.8			12.5 ± 4.2					7.1 ± 2.0		
No		16.7 ± 4.4		0.63	13.3 ± 4.8		0.56			7.6 ± 3.0		0.60
Degree of perineal injuries^c^												
Intact perineum		12.2 ± 5.5			8.6 ± 4.3				7.5 ± 3.7			
First-degree tear		16.0 ± 4.0			11.9 ± 4.2				7.7 ± 2.8			
Second-degree tear		17.5 ± 3.9			14.0 ± 4.5				7.5 ± 2.9			
Third-degree tear		19.3 ± 4.0			16.3 ± 2.1				9.7 ± 2.5			
Fourth-degree tear			<0.01^*^				<0.01^*^					0.65
Episiotomy^b^												
Yes	18.0 ± 3.7				14.3 ± 4.6				7.9 ± 2.9			
No	15.2 ± 4.9		<0.01^*^		11.9 ± 4.7			<0.01^*^	7.1 ± 2.9			0.12
Taking painkillers^b^												
Yes	17.0 ± 4.3				13.6 ± 4.6				7.0 ± 1.7			
No	15.8 ± 5.0		0.13		12.8 ± 4.9			0.27	7.6 ± 2.9			0.73
1 month postpartum; types of neonatal nutrition^b^												
Breast milk									8.1 ± 3.5			
Combination / Formula									7.1 ± 2.0			0.10

^a^Pearson’s correlation coefficients

^b^Student’s t-test

^c^One-way analysis of variance

SD: Standard Deviation; BMI: Body Mass Index

^*^ p<0.05

Table 6 presents the factors associated with difficulty sitting using mixed model for follow-up data. Difficulty sitting score was significantly higher at day 1 and 5 postpartum compared to 1 month postpartum, adjusting for independent variables that included parity, mode of delivery, episiotomy, perineal injury, perineal pain, and maternal age. No episiotomy showed a significant and negative association with difficulty sitting. Perineal pain showed a significant and positive association with difficulty sitting.

**Table 6:** Factors associated with difficulty sitting using mixed model for follow-up data

	*β*	95% confidence interval	*p*-value
Day 1 postpartum (vs. 1 month postpartum)^a^	5.64	(4.60-6.68)	<0.01^*^
Day 5 postpartum (vs. 1 month postpartum)^a^	3.34	(2.57-4.12)	<0.01^*^
Parity; Primiparous (vs. Multiparous)	0.74	(-0.32-1.79)	0.17
Mode of delivery; Spontaneous (vs. Vacuum)	-0.75	(-2.07-0.57)	0.27
Episiotomy; No (vs. Yes)	-1.40	(-2.43--0.36)	0.01^*^
Perineal injuries; No (vs. Yes)	-1.00	(-2.47-0.46)	0.18
Perineal pain (continuous)	0.09	(0.08-0.11)	<0.01^*^
Maternal age (continuous)	-0.02	(-0.12-0.08)	0.65

^a^Bonferroni correction was applied

^*^ p<0.05

Table 7 presents the factors associated with difficulty moving using mixed model for follow-up data. Difficulty moving was significantly higher at day 1 and 5 postpartum compared to 1 month postpartum, adjusting for independent variables that included parity, mode of delivery, episiotomy, perineal injury, perineal pain, and maternal age. Intact perineum showed a significant and negative association with difficulty moving. Perineal pain showed a significant and positive association with difficulty moving.

**Table 7:** Factors associated with difficulty moving using mixed model for follow-up data

	*β*	95% confidence interval	*p*-value
Day 1 postpartum (vs. 1 month postpartum)^a^	6.94	(5.92-7.96)	<0.01^*^
Day 5 postpartum (vs. 1 month postpartum)^a^	3.84	(3.08-4.61)	<0.01^*^
Parity; Primiparous (vs. Multiparous)	0.76	(-0.30-1.82)	0.16
Mode of delivery; Spontaneous (vs. Vacuum)	-0.86	(-2.19-0.47)	0.20
Episiotomy; No (vs. Yes)	-0.78	(-1.82-0.26)	0.14
Perineal injuries; No (vs. Yes)	-1.84	(-3.31--0.37)	0.01^*^
Perineal pain (continuous)	0.09	(0.07-0.10)	<0.01^*^
Maternal age (continuous)	0.01	(-0.09-0.11)	0.89

^a^Bonferroni correction was applied

^*^ p<0.05

Table 8 presents the factors associated with difficulties with excretion and cleanliness using mixed model for follow-up data. Difficulties with excretion and cleanliness score was significantly higher at day 1 and 5 postpartum compared to 1 month postpartum, adjusting for independent variables that included parity, mode of delivery, episiotomy, perineal injury, perineal pain, and maternal age. Primiparous women and perineal pain showed a significant and positive association with difficulties with excretion and cleanliness. Intact perineum showed a significant and negative association with difficulties with excretion and cleanliness.

**Table 8:** Factors associated with difficulties with excretion and cleanliness using mixed model for follow-up data

	*β*	95% confidence interval	*p*-value
Day 1 postpartum (vs. 1 month postpartum)^a^	7.11	(6.16-8.05)	<0.01^*^
Day 5 postpartum (vs. 1 month postpartum)^a^	4.92	(4.23-5.62)	<0.01^*^
Parity; Primiparous (vs. Multiparous)	1.17	(0.05-2.29)	0.04^*^
Mode of delivery; Spontaneous (vs. Vacuum)	-0.28	(-1.68-1.13)	0.70
Episiotomy; No (vs. Yes)	-0.47	(-1.57-0.63)	0.40
Perineal injuries; No (vs. Yes)	-2.34	(-3.87--0.80)	<0.01^*^
Perineal pain (continuous)	0.06	(0.05-0.07)	<0.01^*^
Maternal age (continuous)	0.09	(-0.02-0.19)	0.11

^a^Bonferroni correction was applied

^*^ p<0.05

## Discussion

### Patterns of interference with activities of daily living and perineal pain

Our study clearly showed that interference with activities of daily living and perineal pain decreased from day 1 to 5 postpartum and day 5 to 1 month postpartum. This trend remained, adjusted for confounders using a linear mixed model.

The rate of episiotomy in our study was higher (82.6% for primiparous and 27.2% for multiparas) than those reported in Australia (23%) [13] or United Kingdom (22%) [14]. This higher prevalence could have affected our findings. East et al.’s Cochrane review reported that women had self-rated perineal pain at one or more of the pre-specified times between four and six hours, within the first 24 hours, or between 24 to 48 hours after giving birth [4]. In addition, perineal pain decreases over time, as well as does the interference with activities of daily living in those with perineal pain [1]. Our results showed a similar trend to previous studies, despite the high episiotomy rate.

In a longitudinal Canadian study with 444 postpartum women (263 primiparous and 181 multiparous women) the prevalence of women with ‘sitting’ interference was 10.5%, 7.6%, and 0.6% at day 1, day 7, and 6 weeks postpartum, respectively. The prevalence of women with ‘urinate’ interference was 1.1%, 3.5%, and 1.4% at day 1, day 7, and 6 weeks postpartum, respectively. In addition, the prevalence of women with ‘walking’ interference was 3.1%, 3.5%, and 0.7% at day 1, day 7, and 6 weeks postpartum, respectively [1]. In the study, women were interviewed regarding interference with daily activities, such as sitting, urinating, and walking, using a single yes/no question. However, we used a composite of elements, such as difficulty sitting, difficulty moving, and difficulties with excretion and cleanliness. Thus, our categories of activities of daily living represented further detailed or complex actions than previous studies [1, 6]. In our study, the prevalence of women with no ‘difficulty sitting’ was 10.9% (n=20/184), 30.4% (n=56/184), and 84.3% (n=102/121) at day 1, day 5, and 1 month postpartum, respectively. Our results showed similar trends to those of previous study, which reported that sitting interference decreased over time, despite different measurement.

### Relevant factors for interference with activities of daily living

Macarther and Macarthur [1] found an association between the degree of perineal injury and interference with activities of daily living; however, they did not adjust for the intensity of perineal pain. In our study, difficulty sitting, difficulty moving, and difficulties with excretion and cleanliness showed an independent association with perineal pain and perineal injury or episiotomy. Takeuchi and Yanai [15] used structural equation modelling and found that three factors disrupted women’s daily life in the early postpartum period: primiparous, perineal trauma, and perineal pain. Imarengiaye and Andet [16] reported that the in relative risk of perineal pain for the perineal trauma group, with no trauma group as the reference group, sitting interference was consistently the most affected on day 1 and 3 postpartum. These previous studies reported similar results to ours that perineal injuries and/or episiotomy and perineal pain clearly affected interference with activities of daily living.

### Implications for practice

Our results could show a significant association between perineal pain and interference with activities of daily living from day 1 to 1 month postpartum. Persistent pain or discomfort in the perineum had an impact on a woman’s ability to taking care of her new baby [17] and negative emotional well-being [18] and lead to long-term effects, such as sexual and pelvic dysfunction [19]. Therefore, reducing pain and discomfort in the early postpartum period and minimising long-term complications can improve postpartum women’s physical and mental health.

First, additional attention is required to reduce perineal pain and avoid unnecessary perineal injury for less interference with activities of daily living in the postpartum period, especially in areas where high episiotomy rates are estimated, such as low-and-middle income countries and Japan. Therefore, midwives need to be actively involved to reduce perineal pain in the postpartum period, such as cold perineal compresses [4], and advising primiparous women to maintain gestational weight gain within the recommended range [20]. Furthermore, midwives should provide guidance to help women engage in perineal care, such as perineal massage [21, 22] and warm perineal compresses [23], which may be useful in reducing perineal injury during delivery.

### Limitations

Our study has some strengths and limitations. We reported an independent association between perineal pain and delivery-related factors with interference with activities of daily living. Regarding limitations, our study included only Japanese women at term. Therefore, our results may not be applicable to pregnant women with complications and women in occidental countries. Second, our study did not include the effect of timing of painkiller taken by the participants on the perineal pain self-rating, which might have affected the intensity of perineal pain. Third, owing to the small sample size, we were unable to analyse the degree of perineal injury. Thus, we were unable to further investigate the association with interference with activities of daily living. Further studies are required from the perspective of improving postpartum women’s quality of life.

## Conclusion

Perineal pain and interference with activities of daily living, which included difficulty sitting, difficulty moving, and difficulties with excretion and cleanliness, reduced from day 1 to 5 postpartum and further from day 5 to 1 month postpartum. Positive relationships were observed between perineal pain and interference with activities of daily living until 1 month postpartum, adjusted for perineal injury and episiotomy, despite the decrease in perineal pain and interference with activities of daily living. Women experienced severe perineal pain and interference with activities of daily living at the beginning of rooming-in and child-rearing, especially in the early postpartum period. Therefore, to promote maternal role attainment through child-rearing since early postpartum, midwives should pay additional attention that mothers’ perineal pain as it could negatively affect their daily activities and child-rearing.

## Data Availability

We obtained consent from participants according to the Nagoya University research ethics guidelines, and participants agreed that their data would be stored at Nagoya University for ten years. Therefore, the datasets generated and/or analysed during the current study are not publicly available due to lack of consent from participants to share their raw data in a public repository, but are available from the corresponding author on reasonable request.

## Abbreviations

VAS: Visual analogue scale

## References

[1] Macarthur AJ, Macarthur C (2004). Incidence, severity, and determinants of perineal pain after vaginal delivery: A prospective cohort study. Am J Obstet Gynecol..

[2] Brito APA, Caldeira CF, Salvetti MG (2021). Prevalence, characteristics, and impact of pain during the postpartum period. Rev Esc Enferm USP..

[3] Molin B, Sand A, Berger AK, Georgsson S (2020). Raising awareness about chronic pain and dyspareunia among women – a Swedish survey 8 months after childbirth. Scand J Pain..

[4] East CE, Dorward EDF, Whale RE, Liu JJ (2020). Local cooling for relieving pain from perineal trauma sustained during childbirth. Cochrane Database Syst Rev.

[5] Lai YL, Hung CH, Stocker J, Chan TF, Liu Y (2015). Postpartum fatigue, baby-care activities, and maternal-infant attachment of vaginal and cesarean births following rooming-in. Appl Nurs Res..

[6] Pereira TRC, De Souza FG, Beleza ACS (2017). Implications of pain in functional activities in immediate postpartum period according to the mode of delivery and parity: An observational study. Braz J Phys Ther..

[7] Takeuchi S (2014). Nursing care for postpartum perineal pain at Japanese hospitals and birth centers. Jpn J Matern Health..

[8] World Health Organization. WHO statement on Caesarean section rates; 2015. https://apps.who.int/iris/bitstream/handle/10665/161442/WHO_RHR_15.02_eng.pdf?sequence=1. Accessed 1 Dec 2023.

[9] Ministry of Health, Labour and Welfare. Overview of statistics on births in 2021. Multidimensional analysis of birth trends; 2021. https://www.mhlw.go.jp/toukei/saikin/hw/jinkou/tokusyu/syussyo07/dl/02.pdf. Accessed 1 Dec 2023. (in Japanese).

[10] NAGOYA Repository. http://hdl.handle.net/2237/0002006851. Accessed 1 Dec 2023. (in Japanese). Doctoral thesis; 2023.

[11] Hsieh CH, Chen CL, Han TJ, Lin PJ, Chiu HC (2018). Factors influencing postpartum fatigue in vaginal-birth women: Testing a path model. J Nurs Res..

[12] Huskisson EC (1974). Measurement of pain. Lancet..

[13] Australian Institute of Health and Welfare. Australia’s Mothers and Babies. https://www.hqip.org.uk/resource/national-maternity-and-perinatal-audit-nmpa-clinical-report-2019/. Accessed 1 Dec 2023, 2020; 2018—in brief.

[14] Healthcare Quality Improvement Partnership. National Maternity and Perinatal Audit (NMPA) clinical report. https://maternityaudit.org.uk/FilesUploaded/NMPA%20Clinical%20Report%202019.pdf. Accessed 1 Dec 2023, 2019; 2019.

[15] Takeuchi S, Yanai H (2013). Factors associated with perineal pain and effect of daily life during early postpartum. J Jpn Acad Nurs Sci..

[16] Imarengiaye CO, Andet AB (2008). Postpartum perineal pain among Nigerian women. West Afr J Med..

[17] East CE, Sherburn M, Nagle C, Said J, Forster D (2012). Perineal pain following childbirth: Prevalence, effects on postnatal recovery and analgesia usage. Midwifery..

[18] Subki AH, Fakeeh MM, Hindi MM, Nasr AM, Almaymuni AD, Abduljabbar HS (2019). Fecal and urinary incontinence associated with pregnancy and childbirth. Mater Sociomed..

[19] Manresa M, Pereda A, Bataller E, Terre-Rull C, Ismail KM, Webb SS (2019). Incidence of perineal pain and dyspareunia following spontaneous vaginal birth: A systematic review and meta-analysis. Int Urogynecol J..

[20] Yamada A, Takahashi Y, Hirose M, Usami Y, Maruya S, Tamakoshi K (2024). Factors associated with perineal pain on the first postnatal day after vaginal delivery: A cross-sectional study of primiparous women. Nagoya J Med Sci..

[21] Ugwu EO, Iferikigwe ES, Obi SN, Eleje GU, Ozumba BC (2018). Effectiveness of antenatal perineal massage in reducing perineal trauma and post-partum morbidities: A randomized controlled trial. J Obstet Gynaecol Res..

[22] Beckmann MM, Stock OM (2013). Antenatal perineal massage for reducing perineal trauma. Cochrane Database Syst Rev.

[23] Aasheim V, Nilsen ABV, Reinar LM, Lukasse M (2017). Perineal techniques during the second stage of labour for reducing perineal trauma. Cochrane Database Syst Rev..

